# G-Protein Coupled Estrogen Receptor in Breast Cancer

**DOI:** 10.3390/ijms20020306

**Published:** 2019-01-14

**Authors:** Li-Han Hsu, Nei-Min Chu, Yung-Feng Lin, Shu-Huei Kao

**Affiliations:** 1 Ph.D. Program in Medical Biotechnology, College of Medical Science and Technology, Taipei Medical University, Taipei 110, Taiwan; lhhsu@kfsyscc.org (L.-H.H.); yflin@tmu.edu.tw (Y.-F.L.); 2 Division of Pulmonary and Critical Care Medicine, Sun Yat-Sen Cancer Center, Taipei 112, Taiwan; 3 Department of Medicine, National Yang-Ming University Medical School, Taipei 112, Taiwan; 4 Department of Medical Oncology, Sun Yat-Sen Cancer Center, Taipei 112, Taiwan; nmchu@kfsyscc.org; 5 School of Medical Laboratory Science and Biotechnology, College of Medical Science and Technology, Taipei Medical University, 250 Wu-Hsing Street, Taipei 110, Taiwan

**Keywords:** breast cancer, epidermal growth factor receptor, estrogen, estrogen receptor, G-protein coupled estrogen receptor

## Abstract

The G-protein coupled estrogen receptor (GPER), an alternate estrogen receptor (ER) with a structure distinct from the two canonical ERs, being ERα, and ERβ, is expressed in 50% to 60% of breast cancer tissues and has been presumed to be associated with the development of tamoxifen resistance in ERα positive breast cancer. On the other hand, triple-negative breast cancer (TNBC) constitutes 15% to 20% of breast cancers and frequently displays a more aggressive behavior. GPER is prevalent and involved in TNBC and can be a therapeutic target. However, contradictory results exist regarding the function of GPER in breast cancer, proliferative or pro-apoptotic. A better understanding of the GPER, its role in breast cancer, and the interactions with the ER and epidermal growth factor receptor will be beneficial for the disease management and prevention in the future.

## 1. Introduction

**Figure 1:**
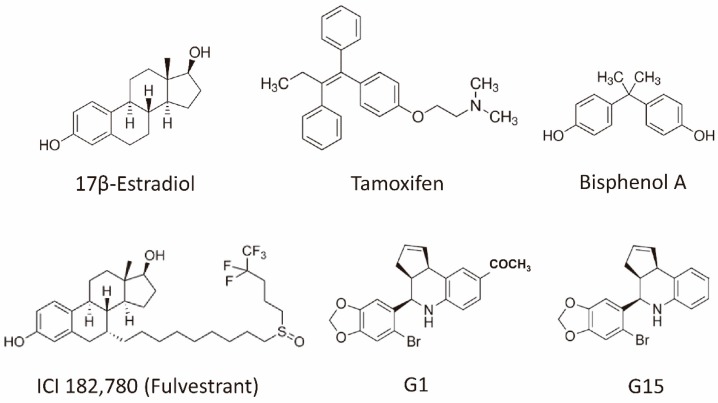
Structures of the representative GPER agonists and antagonists. 17β-estradiol is one of the major physiological forms of estrogen. Tamoxifen is both a selective estrogen receptor modulator and an agonist for the GPER. Bisphenol A is a xenoestrogen. Fulvestrant is a selective estrogen receptor downregulator (ER antagonist) and an agonist for the GPER. G-1 is a selective GPER agonist, whereas G15 is a selective GPER antagonist. Abbreviation: ER, estrogen receptor; GPER, G-protein coupled estrogen receptor.

**Figure 2:**
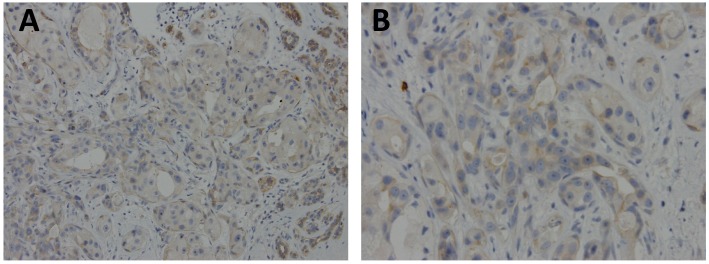
Representative case of archival, paraffin-embedded breast ductal carcinoma stained with polyclonal GPER1 antibody (Sigma-Aldrich, 1:50 dilution) showed focal, weak membranous and cytoplasmic expression. (**A**) original × 200; and (**B**) original × 400.

Breast cancer is the most common cancer among women worldwide [1,2]. There were over two million new cases in 2018. In 2015, 14,801 new cases were diagnosed in Taiwan [3]. It ranked fourth in mortality in Taiwan and led to the death of 18.2 persons per 100,000 of the population. Estrogen, predominantly 17β-estradiol (E_2_) and its receptor has long been known to enhance the development and progression of breast cancer. Drugs targeting the estrogen signaling pathway through the selective estrogen receptor modulator (SERM) (e.g., tamoxifen, raloxifene), the estrogen receptor (ER) antagonists (e.g., fulvestrant) and, the aromatase inhibitors, including the reversible nonsteroidal agents (e.g., letrozole, anastrozole), or the irreversible steroidal inactivator (e.g., exemestane), has been used for decades to treat ER positive breast cancer (Figure 1) [4]. Tamoxifen is the first SERM approved for the treatment of breast cancer and effectively demonstrated in the reduction of the recurrence and prevention of contralateral breast cancer. However, primary or acquired resistance frequently arises and becomes the major obstacle in hormone therapy, which indicates a more complex receptor and signaling pathways involved in the cancer progression. The G-protein coupled estrogen receptor (GPER), originally known as GPR30, a seven transmembrane domain protein, is an alternate estrogen receptor with a structure distinct from the two canonical estrogen receptors, ERα and ERβ mainly mediate a rapid non-genomic response [5,6,7,8,9,10]. This is expressed in about 50% to 60% of breast cancer tissues and has been reported as a modulator of neoplastic transformation (Figure 2) [11,12,13,14,15,16,17,18,19,20,21,22,23,24,25]. Paradoxically, the modulators or antagonists of the classical estrogen receptors such as tamoxifen, raloxifene, and fulvestrant, were found to be the GPER agonists [24]. The expression of GPER has been presumed to be associated with the development of tamoxifen resistance [26,27,28,29,30,31,32]. In breast cancer patients treated with tamoxifen, there is an increased risk of developing endometrial cancer and often has a poor clinical outcome. GPER was also supposed to mediate the contrary tissue-specific effect [33,34]. 

On the other hand, triple-negative breast cancer (TNBC), defined as a lack of ERα, progesterone receptor (PR), or the overexpression of human epidermal growth factor receptor 2 (HER2/neu), constitutes 15% to 20% of breast cancers. It is more prevalent in younger women and is frequently present at a more advanced stage with a more aggressive behavior. Lacking a well-defined receptor and signaling pathway, chemotherapy remains the treatment of choice but with a higher rate of recurrence. GPER is prevalent in TNBC and presumed to be involved in the growth of TNBC. It can be considered as a candidate of therapeutic target [35,36,37,38,39,40]. 

The endocrine disruptive chemicals, such as bisphenol (Figure 1) and thiodiphenol, at the environmentally relevant doses may exert effects through the GPER and estrogen-like signaling pathways, contribute to breast cancer progression, and drug resistance in both the ERα-positive and -negative breast cancer cells [41,42,43,44,45]. 

The epidermal growth factor receptor (EGFR) activation is a common and important event in the pathogenesis and progression of breast cancer. The EGFR transactivation by estrogen via the GPER has been proposed as an alternate signaling pathway with a potential significance for breast cancer [46,47,48,49,50,51,52,53,54].

However, contradictory results have existed regarding the response of GPER to estrogens/antiestrogens and the effect of GPER agonist/antagonist on the proliferation, migration and invasion of the breast cancer cells [55,56,57,58,59,60,61,62,63,64,65]. There were controversies on the subcellular localization of GPER and its function, proliferative or pro-apoptotic. Therefore, the role of GPER in ERα positive breast cancer and TNBC remains unclear. 

In the following, updated evidence about the GPER in breast cancer were examined. Future epidemiology and laboratory studies, which may be helpful to elucidate the role of the GPER, were proposed.

## 2. G-Protein Coupled Estrogen Receptor Expression in Breast Cancer

The significance of GPER in human breast cancer was evaluated by comparing its relationship to ER, PR, and the cancer progression variables through immunohistochemical analysis [15]. A significant association between the GPER and ER was observed. GPER was positively correlated with the HER2/neu expression, tumor size, and metastasis. The distinct patterns of the GPER and ER in association with the cancer progression variable supported that the GPER and ER have independent influences on the estrogen responsiveness of breast cancer. The association between GPER expression and tamoxifen resistance was later confirmed [27]. The GPER was negatively correlated with relapse-free survival in patients only treated with tamoxifen. Multivariate analysis revealed that the GPER expression was an independent prognosticator for a poor outcome. In a study of postmenopausal lymph node negative breast cancer patients, the absence of the plasma membrane GPER predicted a 91% 20-year distant disease-free survival, as compared to 73% in the presence of GPER for the tamoxifen-treated ER-positive and PR-positive subgroup [20]. The GPER overexpression and plasma membrane localization are critical events in breast cancer progression. GPER was also prevalent in the TNBC, and the GPER expression was associated with a younger age and a more aggressive disease [37].

As compared with the corresponding primary tumors in the same patients, GPER expression in the recurrent tumors or metastases significantly increased under the tamoxifen treatment [27,28].

The tissue microarrays from the formalin-fixed, paraffin embedded samples of the primary invasive breast carcinomas suggested that the predominantly cytoplasmic or nuclear GPER expression were two distinct immunohistochemical patterns and may reflect different biological features [19]. Cytoplasmic GPER expression was associated with non-ductal histology, lower stage, more differentiation, and better overall survival, whereas the nuclear GPER expression was associated with poor differentiation and TNBC. In the breast cancer cell lines, confocal microscopy revealed the different GPER expression patterns. The T47D cells had a strong GPER expression, predominantly localized in the cytoplasm. The MCF-7 cells showed a less strong GPER expression and a mainly nuclear distribution. No distinct plasma membranous expression was observed.

Briefly, GPER was prevalent in the ERα positive breast cancer and TNBC. The GPER was a prognosticator for a poor outcome. There was a higher GPER expression in the re-biopsy specimen of tamoxifen resistant ERα positive breast cancer and chemotherapy refractory TNBC than the primary tumor. It should be noted that the subcellular location of the GPER may have a different prognostic implication in breast cancer.

## 3. G-Protein Coupled Estrogen Receptor Functions in Breast Cancer

**Figure 3:**
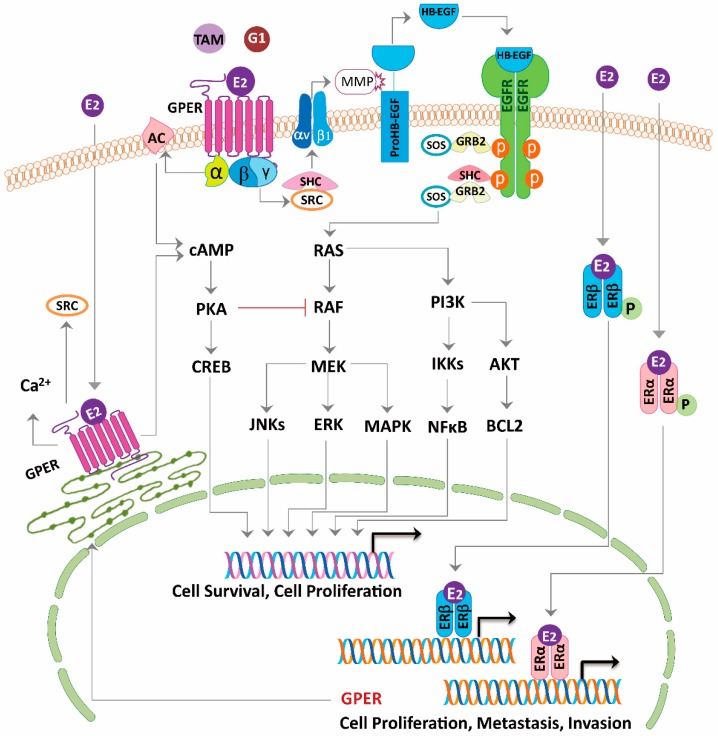
Model of estrogen signaling pathways in cancer. 17β-estradiol (E_2_) activates ERα or ERβ to induce the receptor dimerization, and subsequently acts as a transcription factor or interacts with other transcription factors binding to the promoter region of the target genes. E_2_, tamoxifen (TAM) or G1 activate the G-protein Coupled Estrogen Receptor (GPER) distributed in the nucleus, cytoplasm, and plasma membrane. Activation of GPER located in the plasma membrane stimulates steroid receptor coactivator (SRC) through a Gβγ-subunit protein pathway. The β and γ subunits of the G protein activate the SRC tyrosine kinase, which binds to the integrin ανβ1 through the SHC adapter protein. The complex activates the matrix metalloproteinase (MMP), which then cleaves the pro-heparin-binding EGF-like growth factor (proHB-EGF) and releases the heparin-binding EGF-like growth factor (HB-EGF) into the extracellular space. The free HB-EGF then transactivates the epidermal growth factor receptor (EGFR). Phosphorylation of EGFR in turn activates the downstream pathways, which can induce rapid non-genomic effects, or genomic effects regulating different genes transcription and leads to cell survival and proliferation. On the other hand, through GPER, E_2_, tamoxifen or G1 is able to stimulate the adenylyl cyclase activity through a Gα-subunit protein pathway, which then leads to the protein kinase A (PKA)-mediated suppression of the EGFR-induced ERK activity. Thus, via the GPER, E_2_, tamoxifen or G1 may balance the ERK activity by stimulating two distinct G-protein signaling pathways that have opposing effects on the EGFR-to-MAPK axis. Long-term tamoxifen treatment could sensitize the cancer cells through E_2_-stimulated upregulation of GPER and translocation from the endoplasmic reticulum to the plasma membrane.

The 17β-estradiol activated the extracellular regulated protein kinase 1/2 (ERK1/2), not only in the ERα-positive and the ERβ-positive MCF-7 cells, but also in the ERα-negative and the ERβ-negative SkBr3 cells [47]. Immunoblot analysis showed that this estrogen response was associated with the presence of the GPER protein in these cells. The ERα-negative, ERβ-positive MDA-MB-231 cells are GPER deficient and insensitive to ERK1/2 activation by E_2_. Transfection of the MDA-MB-231 cells with a GPER complementary DNA resulted in the overexpression of a GPER protein and conversion to an estrogen-responsive phenotype. In addition, the GPER-dependent ERK1/2 activation could be triggered by the ER antagonist, fulvestrant. The E_2_ signaling to the ERK1/2 occurred via a Gβγ-dependent, pertussis toxin-sensitive pathway. The β and γ subunits of the G protein activate the steroid receptor coactivator (SRC) tyrosine kinase, which binds to the integrin ανβ1 through the SHC adapter protein (Figure 3). The complex activates the matrix metalloproteinase, which then cleaves the pro-heparin-binding EGF-like growth factor and releases the heparin-binding EGF-like growth factor (HB-EGF) into the extracellular space. The free HB-EGF then transactivates the EGFR. The E_2_ signaling to the ERK1/2 could be blocked by down-modulating the HB-EGF from the cell surface with the diphtheria toxin mutant, CRM-197, neutralizing the HB-EGF with antibodies, or inhibiting the EGFR tyrosine kinase activity. ER-negative breast cancers that continue to express GPER may use estrogen to drive the EGFR-dependent cellular responses [47,50,51]. The crosstalk between the GPER and the EGFR was confirmed in the tamoxifen-resistant ERα positive cell, TNBC, and cancer-associated fibroblast (CAF), respectively (Table 1) [26,28,38,66,67].

### 3.1. Tamoxifen-Resistant ERα Positive Cells 

The tamoxifen-resistant cells, TAM-R, exhibited an enhanced sensitivity to the E_2_ and the GPER-specific agonist, G1 (Figure 1), when compared to the parental MCF-7 cells [26,28]. Tamoxifen was able to stimulate the mitogen-activated protein kinase (MAPK) phosphorylation and cell growth in the TAM-R cells, and the effects were abolished by the GPER antagonist, G15 (Figure 1), the GPER anti-sense oligonucleotide, the selective SRC inhibitor PP2, and the EGFR inhibitor AG1478. The basal EGFR expression was only slightly elevated in the TAM-R cells, and the basal GPER expression, phosphorylation of the Ak strain transforming murine thymoma viral oncogene (AKT), and the MAPK remained unchanged when compared to the parental cells. Continuous treatment of the MCF-7 cells with G1 mimics the long-term treatment with tamoxifen and drastically increases its agonistic activity. Interestingly, the estrogen treatment significantly increased the GPER plasma membrane translocation, which was stronger in the TAM-R cells. The GPER plasma membrane translocation facilitated the crosstalk with the EGFR. The results have suggested the importance of the GPER and EGFR transactivation in the development of tamoxifen resistance. Combined therapy with the G15 and tamoxifen promoted apoptosis in a TAM-R xenograft and inhibited the drug-resistant tumor progression. The GPER activation led to the nuclear translocation of the forkhead box O3a, FOXO3a, and the down-regulation of caspase 3 and caspase 7 via the phosphoinositide 3-kinase (PI3K)/AKT pathway in the MCF-7 cells [52].

### 3.2. Triple-Negative Breast Cancer Cells

The GPER was strongly expressed in the TNBC cell lines, MDA-MB-468, and MDA-MB-436 [38]. Treatment with E_2_, tamoxifen, and the GPER-specific agonist, G1 led to the rapid activation of ERK1/2, but not AKT. The estrogen/GPER/ERK signaling pathway was involved in the increased cell growth, survival, migration, and invasion through upregulating the expression of cyclin A, cyclin D1, Bcl-2, and c-fos that were associated with the cell cycle, anti-apoptosis, and proliferation, respectively. Pretreatment with the GPER antagonist, G15, AG1478, the ERK1/2 inhibitor, U0126, or the transfection with the siRNA against the GPER could abolish the effects. Immunohistochemical analysis of the TNBC specimens showed a significantly stronger staining of the p-ERK1/2 in the GPER-positive tissues than the GPER-negative tissues [38]. The positivity of the GPER and p-ERK1/2 displayed a strong association with the large tumor size and advanced stage, indicating that the GPER/ERK signaling might also contribute to the tumor progression in the TNBC patients, which correlated with the in vitro experimental results. 17β-estradiol and 4-hydroxytamoxifen also increased the proliferation of another two TNBC cell lines, MDA-MB-435 and HCC1806, which was completely prevented by being transfected with siRNA against the GPER [36]. The increased activity of the SRC kinase, EGFR transactivation, and c-fos expression, was also abolished after the knock-down of the GPER expression.

### 3.3. Cancer-Associated Fibroblasts

The GPER was expressed in the stromal fibroblasts of the primary breast cancer tissues, and the CAFs isolated [66,67]. Tamoxifen, in addition to E_2_ and the G1 activated GPER, resulted in the transient increases in the cell index, intracellular calcium, ERK1/2 phosphorylation, and promoted the CAF cell cycle progression, proliferation, and migration. These effects were blocked by the G15, AG1478, and U0126. Importantly, tamoxifen, as well as G1, increased the E_2_ production in the breast CAFs via the GPER/EGFR/ERK signaling pathway when the substrate of E_2_, testosterone, was added to the medium. The GPER-mediated CAF-dependent estrogenic effects in the tumor-associated stroma are more likely to contribute to breast cancer progression, especially in the tamoxifen resistance, via a positive feedback loop involving the GPER/EGFR/ERK signaling pathway and E_2_ production.

17β-estradiol and G1 triggered the GPER/EGFR/ERK/c-fos signaling pathway that led to an increased vascular endothelial growth factor (VEGF) via the upregulation of the hypoxia-inducible factor-1α (HIF1α) in the ER-negative breast cancer cells and the CAFs [53]. The conditioned medium from the CAFs treated with E_2_ and G1 promoted the human endothelial tube formation in a GPER-dependent manner. In the mice breast cancer xenograft model, GPER activation enhanced the tumor growth and the expression of HIF1α, VEGF, and the endothelial marker, CD34. Fatty acid synthase catalyzes the de novo biogenesis of the fatty acids and acts as a metabolic oncogene. 17β-estradiol and G1 regulated the fatty acid synthase expression and activity through the GPER/EGFR/ERK/c-fos/activator protein 1 (AP-1) signaling pathway in the SkBr3 cells and CAFs [54]. 

In summary, E_2_, tamoxifen, and G1 upregulate estrogen production, increase GPER expression and plasma membrane translocation, and stimulate the proliferation, migration, invasion of breast cancer cell lines, and cancer-associated fibroblasts. Pretreatment with the GPER antagonist, G15 or transfected with siRNA against GPER attenuates the effects. The tumor promoting effects of the GPER operate through the EGFR transactivation and related signaling pathways.

### 3.4. Controversies about G-Protein Coupled Estrogen Receptor Function in Breast Cancer

Contrary to the majority of studies that have reported a tumor promoting effect of GPER activation in breast cancer, several studies have demonstrated that GPER functions as a tumor suppressor and induces apoptosis (Table 1) [55,56,57,58,59,60,61,62,63,64,65].

The GPER expression by immunohistochemistry had been reported as a prognosticator for the increased distant disease-free survival in patients with ER-positive breast cancer treated with tamoxifen [55]. A constitutive GPER-dependent pro-apoptotic signaling was proposed. The GPER expression at mRNA levels was significantly down-regulated in both the ERα-positive and ERα-negative breast cancer tissues in comparison with their matched normal tissues, and significantly lower in tumor tissues from the patients who had lymph node metastasis than those without [56]. The tumor samples from 118 Taiwanese patients with infiltrating ductal carcinoma of the breast had a lower GPER expression at the mRNA level than that in non-tumor mammary tissues [57]. The correlation of the GPER expression with clinical parameters and patient survival was not significant.

Filardo et al., suggested that via the GPER, estrogens as well as antiestrogens, were capable of stimulating the adenylyl cyclase activity and increasing the cAMP concentration, which in turn, led to the PKA-mediated suppression of the EGFR induced ERK1/2 activity (Figure 3) [58]. Thus, via the GPER, estrogen may balance ERK1/2 activity by stimulating two distinct G-protein signaling pathways that have opposing effects on the EGFR-to-MAPK axis. The other study concurred with the observation that the reduced cAMP generation attenuated the inhibition of EGFR signaling [28]. 

The GPER functions promoted the SkBr-3 but inhibited the MCF-7 cellular proliferation. An ER- and [Ca^2+^]-dependent negative feedback was proposed for the difference [59]. 17β-estradiol is known to downregulate the ERα expression in the MCF-7 cells as a negative feedback regulatory loop to prevent overexpression. Likewise, the GPER may also be negatively regulated by E_2_ via the ER to prevent an excessive GPER-dependent activity, such as being aberrantly high [Ca^2+^]. The maximum increases in [Ca^2+^] were much larger in the SkBr-3 cells than in the MCF-7 cells. It is possible that this was due to the lack of ERs in the SkBr-3 cells, which translated into a lack of negative feedback regulation.

Proliferative results were observed with two non-specific GPER agonists, estrogen and tamoxifen, but not with its specific agonist, G1. The G1-induced inhibitory effect was specific for the GPER expressing cells [60,61]. Radiation induced different changes in the GPER expression among the breast cancer cells; upregulated in the MDA-MB-231 and the MDA-MB-468 cells and down-regulated in the MCF-7 cells. They proposed that it was linked to the form of the p53 protein expressed in each cell. While the MDA-MB-231 and the MDA-MB-468 cells express a non-functional p53 gene, the MCF-7 cells have normal wild-type p53. A feedback mechanism exists between the expression of p53 and GPER. The GPER activation increases the p53 expression, which in turn down-regulates the expression of the GPER [61].

The activation of the GPER suppresses the epithelial mesenchymal transition, migration, and angiogenesis of the TNBC via the nuclear factor-kappa B (NF-κB) signals [62,63]. The GPER also inhibits the tumor necrosis factor alpha-induced expression of interleukin 6 through the repression of the NF-κB promoter activity in the SkBr3 cells [64].

Further studies are therefore necessary to define the role of the GPER, proliferative or pro-apoptotic in breast cancer.

## 4. G-protein Coupled Estrogen Receptor Knockout Mice

In contrast with the pharmacological methods, the GPER knockout more clearly understands the GPER function through targeted gene deletion or disruption [17]. Four GPER knockout mice have been reported [68,69,70,71]. Among them, three have the whole GPER coding region deleted, and the fourth with the C-terminal portion of the GPER remaining. Although the GPER was also distributed in normal breast tissues, histopathological analysis did not reveal any abnormalities in the GPER-knockout mice [68]. Mammary gland responses after estradiol with/without progesterone treatment were also unimpaired in the GPER knockout mice. The GPER knockout mice did not show overt phenotypes in viability or reproductive function, but some functions of estrogen have been absent in the GPER knockout mice that support the GPER as a physiologically relevant estrogen receptor [17,68,69,70,71,72]. In the only study of breast cancer, the GPER knockout in the polyoma middle T antigen-mouse mammary tumor virus transgenic mice revealed smaller tumors and reduced metastasis [18]. 

## 5. Interactions between Cancer Cells and Cancer-Associated Fibroblasts through G-Protein Coupled Estrogen Receptor

An increased aromatase expression and activity was found in the tamoxifen resistant breast cancer cells [30]. Knocking-down the GPER expression reversed the enhanced aromatase levels. In the ER-negative, GPER/aromatase-positive SkBr3 cells, tamoxifen acted as a GPER agonist. The tamoxifen treatment increased the aromatase expression through an enhanced recruitment of the c-fos/c-jun complex to the AP-1 responsive elements located within the promoter region. Tamoxifen also induced aromatase expression via the GPER in the CAFs. The increased estrogen production in the microenvironment may well lead to a more aggressive behavior of breast cancers.

On co-culturing the CAFs with the breast cancer cells, a significant GPER translocation from the nucleus to the cytoplasm was observed in the CAFs, similar to that observed in the stromal fibroblast in the breast cancer tissues, indicating that the cancer cells may affect the subcellular localization of the GPER in the CAFs. CRM1, a nuclear export protein, and activated PI3K/AKT signaling pathway are involved in the cytoplasmic GPER translocation in the CAFs, which in turn activates a novel estrogen/GPER/cyclic AMP (cAMP)/protein kinase A (PKA)/cAMP response element binding protein signaling pathway, and triggers aerobic glycolysis in the CAFs [73]. The glycolytic CAFs feed pyruvate and lactate to cancer cells to undergo oxidative phosphorylation and contribute to drug resistance. The stromal GPER-mediated drug resistance from the reprogramming of the tumor energy metabolism, i.e. the “reverse Warburg effect”, provided the rationale for the CAFs as a promising target for therapy [74,75]. The different subcellular location of the GPER in the breast CAFs may have biological implications [76]. Targeting the cytoplasmic GPER in the CAFs may restore the response to treatments in both the ER-positive and -negative breast cancers.

## 6. Future Perspectives in the Study of the G-Protein Coupled Estrogen Receptor in Breast Cancer

The inconsistent observations among these studies could be attributed to the usage of the different subtypes of breast cancer samples and the different subcellular localization of the GPER [60], the difference of the cell types and treatment conditions, and the specificities of the agonist [61]. The specificity of the GPER antibodies used in the immunohistochemistry and Western blot may affect the staining patterns of the GPER [77]. The epigenetic of GPER, tumor microenvironment, and hormone levels also affected the results [65]. 

Systemic approaches via epidemiology and laboratory studies are necessary to confirm the role of GPER in breast cancer in the future. ERα positive breast cancer and TNBC need to be studied separately. What is the true prevalence of GPER in the ERα positive breast cancer and the TNBC, respectively? The GPER expression of the archival tissue in both tumor and associated stroma should be measured by a unified scoring system including the staining intensity and the proportion of positive cancer cells, as per the Allred scoring for ER [78]. The subcellular location of the GPER, i.e. nucleus, cytoplasm, and plasma membrane, also need be notified to clarify the implication of the translocation and its role in the prognosis. 

The GPER expression needs to be compared between the tumors and their matched normal tissues. The association of GPER with the ERα, PR, HER2/neu, and the correlations with the clinic-pathologic variables and survival also need to be investigated. In addition to stage, histology, and ER, PR, HER2/neu status, the smoking history, and menopausal status should be included in a multivariate survival analysis to determine as to whether the GPER is an independent prognosticator. Does high or low GPER expression before treatment predict the development of tamoxifen resistance or refractory TNBC? Pairwise comparison of the GPER expression by immunohistochemistry between the archival tissues of the treatment-naïve and re-biopsy specimen of tamoxifen resistant ERα positive breast cancer or the chemotherapy refractory TNBC, will help to better understand whether the GPER becomes predominant during treatment.

The baseline GPER expression in the ERα positive, tamoxifen-resistant ERα positive, TNBC cell lines, and the CAFs, their differences including the subcellular location, the estrogen production including the aromatase activity, the subsequent GPER expression, and the effects, i.e. proliferation, migration, invasion after treatment with E_2_, tamoxifen and G1, and whether the effects are abolished by pretreatment with G15 or siRNA against the GPER will help to confirm the role of GPER. The differences between the effects of E_2_, tamoxifen and G1 should also be observed for the receptor specificity. Immunofluorescent microscopy may be used to observe the cell surface translocation of the GPER, which facilitates the crosstalk with EGFR. In addition to the ERK signaling pathway, is the AKT pathway involved in the GPER-EGFR transactivation [38]? The reciprocal changes of the GPER expression, the biomarkers for the “reverse Warburg effect”, and the lactate shuttle such as the mono-carboxylate transporters 4, and the mitochondrial activities in the cancer cell lines and the CAFs, on treatment with E_2_, tamoxifen, or G1 also deserve to be studied, to explore the role of the metabolic coupling that occurs between the CAFs and the cancer cells.

For the reported anti-proliferative and pro-apoptotic effects of the GPER activation in the literature, the stimulation of the adenylyl cyclase to increase the intracellular cAMP or intracellular Ca^2+^ mobilization as a second messenger, and the pro-apoptotic signaling, measured by the increased cytochrome C release, caspase-3 cleavage, poly(ADP-ribose) polymerase, PARP cleavage, and the decreased cell viability after treatment with estrogen, tamoxifen, G1, or G15, could be correlated to understand the regulatory mechanism [58]. In contrast with the chronic exposure at a low nanomolar dosage, the higher bisphenol concentration in the micromolar exerts an anti-proliferative effect on the cancer cells in spite of the activation of the EGFR/ERK signaling pathway. An increased expression of p53 and its phosphorylation was observed. Therefore, we need to investigate whether the GPER effects are modified by the simultaneous p53 activation [79]. 

Several other signaling pathways, such as HIPPO, NOTCH, and target genes and proteins, such as SNAIL, β1-integrin, focal adhesion kinase, calpain, and connective tissue growth factor, have been reported to be involved in the GPER-mediated breast cancer progression [29,80,81,82,83]. The microRNAs target numerous genes and are involved in cancer progression. GPER is an important regulator of microRNAs. In the MCF-7 and MDA-MB-231 cells, the GPER activation down-regulated the miR-148a and caused an increase in the human leukocyte antigen-C expression that led to the cancer cells escaping from immune surveillance and allowed cancer progression [84]. In the SkBr-3 cells and CAFs, the GPER activation up-regulated the miR-144, which in turn inhibited the tumor suppressor runt-related transcription 1 factor, and increased the cell cycle propagation [85]. Systemic studies of microRNA expression are needed to better define the regulations by the GPER and their effects.

To include GPER in the functional screens for genes contributing to tamoxifen resistance in breast cancer cells and the usage of novel technology, e.g., DNA microarray, and proteomic analysis may help to investigate the association [86,87]. Insulin-like growth factor-I and HIF1α were shown to increase the GPER expression in the breast cancer cells and led to cell proliferation, migration, and tumor angiogenesis [88,89]. The epidermal growth factor may reciprocally up-regulate the GPER to facilitate a stimulatory role of estrogen, even in the TNBC [40]. How the GPER expression is regulated, e.g., through epigenetic by methylation or demethylation of the promoter, requires further studies to understand the EGFR and GPER crosstalk [61,90]. 

The classical ERs do not contain a hydrophobic part that may serve as a transmembrane domain. However, the presence of the ERs in the membrane of the somatic and cancer cells, and the rapid non-genomic responses that occur through the membrane-bound classical ERα and ERβ have been reported [49,91,92,93]. How the classical ERs translocate to the membrane [94], and the interactions between the classical ERs and the GPER is important to understand the membrane-associated non-genomic pathways of estrogen. 

## 7. Conclusions

In this review, we have tried to explore the actions and to understand the molecular basis of the agonist/antagonist mechanisms of the GPER in breast cancer with tamoxifen resistance, and TNBC from the current epidemiology and laboratory studies. The combined non-genomic and genomic effects of estrogen are critical for its overall function, even in the absence of ligand, and the interactions between multiple receptors are complex [95]. Further studies will help to clarify the role of the GPER and support it as a novel target of therapeutic strategies. GPER expression may also be valuable as prognostic or predictive biomarkers.

## Figures and Tables

**Table 1:** G-protein coupled estrogen receptor (GPER) as a prognosticator in breast cancer cell lines and tissues.

References	Materials	Methods	Subcellular Localizations	Effects on Tumor
**Tamoxifen-resistant ERα positive cells**				
Ignatov 2010 [26]	MCF-7, TAM-R MCF-7	Western blot	membrane/endoplasmic reticulum	promoting
Ignatov 2011 [27]	TAM-R cancer tissue	immunohistochemistry	nucleus/cytoplasm	promoting
Mo 2013 [28]	MCF-7, TAM-R MCF-7	immunohistochemistry	membrane/cytoplasm	promoting
	TAM-R cancer tissue	immunofluorescence		
	TAM-R mouse xenograft	RT-PCR, Western blot		
Chen 2014 [29]	MCF-7, SkBr3 cells	qRT-PCR, Western blot	non-specified	promoting
Catalano 2014 [30]	MCF-7, TAM-R MCF-7, SkBr3, CAF	RT-PCR, Western blot for aromatase activity	non-specified	promoting
**Triple-negative breast cancer cells**				
Lappano 2010 [35]	MCF-7, SkBr3	RT-PCR, Western blot	non-specified	promoting
Girgert 2012 [36]	MDA-MB-435, HCC1806	RT-PCR, Western blot	non-specified	promoting
Steiman 2013 [37]	TNBC cancer tissue	immunohistochemistry	non-specified	promoting
Yu 2014 [38]	MDA-MB-468, MDA-MB-436	immunohistochemistry	nucleus/cytoplasm	promoting
	TNBC cancer tissue	immunofluorescence		
		RT-PCR, Western blot		
Zhou 2016 [39]	SkBr3, MDA-MB-231	Western blot	nucleus/cytoplasm	promoting
		immunofluorescence		
Albanito 2008 [40]	SkBr3, BT20	RT-PCR, Western blot	nucleus/cytoplasm	promoting
		immunofluorescence		
**Cancer-associated fibroblast**				
Luo 2014, 2016 [66,67]	CAFs isolated from surgical	RT-PCR, Western blot	nucleus/cytoplasm	promoting
	specimens	immunofluorescence		
**Conflicting results**				
Broselid 2013 [55]	ER-(+) cancer tissue	RT-PCR, Western blot	non-specified	suppressive
	MCF-7 ± GPER knockdown	immunofluorescence		
	T47D, HEK ± GPER			
Poola 2008 [56]	ER-(+)&(−) cancer tissue	qRT-PCR	non-specified	suppressive
Kuo 2007 [57]	ER-(+)&(−) cancer tissue	qPCR	non-specified	suppressive
Filardo 2002 [58]	MCF-7, SkBr3, MDA-MB-231	Western blot	non-specified	suppressive
Ariazi 2010 [59]	ER-(+)/(−) cancer microarray	RT-PCR, Western blot	non-specified	suppressive
	MCF-7, SkBr3	Ca^2+^ imaging		
Weißenborn 2014 [60]	MCF-7, SkBr3	RT-PCR, Western blot	non-specified	suppressive
		methylation PCR		
		bioinformatic		
Weißenborn 2014 [61]	MDA-MB-231, MDA-MB-468	RT-PCR, Western blot	non-specified	suppressive
		methylation PCR		
		bioinformatic		
Chen 2016 [62]	MDA-MB-231	qRT-PCR, Western blot	nucleus/cytoplasm	suppressive
	TNBC cancer tissue	immunofluorescence		
	MDA-MB-231 mice xenograft			
Liang 2017 [63]	MDA-MB-231	qRT-PCR, Western blot	nucleus/cytoplasm	suppressive
	TNBC tissue microarray	immunofluorescence		
	MDA-MB-231 mice xenograft			
Okamoto 2016 [64]	SkBr3 cells	qRT-PCR, Western blot	non-specified	suppressive

ER, estrogen receptor; TAM-R, tamoxifen-resistant; TNBC, triple-negative breast cancer; CAF, cancer-associated fibroblast; qRT-PCR, quantitative RT-PCR.

## Abbreviations

AKT	Ak strain transforming murine thymoma viral oncogene
AP-1	activator protein 1
CAF	cancer-associated fibroblast
cAMP	cyclic AMP
E_2_	17β-estradiol
EGFR	epidermal growth factor receptor
ER	estrogen receptor
ERα	estrogen receptor α
ERβ	estrogen receptor β
ERK	extracellular regulated protein kinase
GPER	G-protein coupled estrogen receptor
HB-EGF	heparin-binding EGF-like growth factor
HER2/neu	human epidermal growth factor receptor 2
HIF1α	hypoxia-inducible transcription factor-1α
MAPK	mitogen-activated protein kinase
NF-κB	nuclear factor-kappa B
PI3K	Phosphoinositide 3-kinase
PKA	protein kinase A
PR	progesterone receptor
SERM	selective estrogen receptor modulator
SRC	steroid receptor coactivator
TNBC	triple-negative breast cancer
VEGF	vascular endothelial growth factor
